# Nanocomposite Cellulose Fibres Doped with Graphene Oxide and Their Biocidal Properties

**DOI:** 10.3390/polym13020204

**Published:** 2021-01-08

**Authors:** Tobiasz Maksymilian Gabryś, Beata Fryczkowska, Alicja Machnicka, Tadeusz Graczyk

**Affiliations:** 1 Department of Material Science, Faculty of Materials, Civil and Environmental Engineering, University of Bielsko-Biala, Willowa 2, 43-309 Bielsko-Biala, Poland; tgraczyk@ath.bielsko.pl; 2 Department of Environmental Protection and Engineering, Faculty of Materials, Civil and Environmental Engineering, University of Bielsko-Biala, Willowa 2, 43-309 Bielsko-Biala, Poland; bfryczkowska@ath.bielsko.pl (B.F.); amachnicka@ath.bielsko.pl (A.M.)

**Keywords:** graphene oxide, cellulose, composite fibres, biocidal properties, zeta potential, isoelectric point

## Abstract

The paper presents a method of obtaining composite cellulose fibres (CEL) doped with graphene oxide (GO) and the influence of GO nanoparticles on the structure and properties of the obtained fibres. Composite fibres (GO/CEL) were prepared using wet method from 5% CEL solutions in 1-ethyl-3-methylimidazolium acetate (EMIMAc) containing GO (0; 0.21; 0.50; 0.98; 1.97% *w/w*) dispersion in N,N-dimethylformamide (DMF). The fibres were coagulated in distilled water and methanol. Optical microscopy allowed us to demonstrate a good degree of GO additive dispersion in the CEL matrix. Surface morphology was examined by scanning electron microscopy (SEM) and infrared spectroscopy (FTIR), which indicated interactions between the matrix and the additive. Strength tests have shown that GO/CEL fibres are characterised by high values of elongation at break (7.7–19.5%) and tenacity (~133–287 [MPa]). The obtained composite fibres are characterized by good biocidal properties against Gram-negative bacteria (*Escherichia coli*), Gram-positive bacteria (*Staphilococcus aureus*), and fungi *Candida albicans*, and the resistance to microorganisms depends on the surface zeta potential value and the isoelectric point (IEP) of GO/CEL fibres.

## 1. Introduction

Natural cellulose fibres are produced by the world of plants (e.g., cotton, sisal, jute, hemp). As a result of chemical treatment of wood cellulose, fibres from regenerated cellulose are obtained. There are many cellulose dissolution systems that can dissolve this biopolymer. Some of them include: N-methylmorpholine-N-oxide (NMMO), ionic liquids (ILs), LiCl/N,N-dimethylacetamide (LiCl/DMAc), NaOH aqua solution, alkali/urea and NaOH/thiourea aqueous solution, tetrabutyl ammonium fluoride/dimethyl sulfoxide, metal complex solutions, and molten inorganic salt hydrates [1]. Woven, knitted and non-woven fabrics are made of cellulose fibres and are used for everyday textile products. The hydrophilic and porous structure of cellulose stimulates the attachment and growth of pathogenic bacteria. Therefore, extensive research is being carried out to impart antibacterial properties to cellulose. The simplest method is surface modification of the fibres. Another method is incorporation of biocidal additives into the spinning liquids (in situ) and then forming composite fibres. Ibrahim et al. applied nanoparticles of metal oxides-ZrO, ZnO, and TiO_2_ onto the surface of cotton fibres [2], while another team introduced CuO into bacterial cellulose [3]. Other researchers introduced silver nanoparticles in the process of cotton mercerization [4]. Lakshmanan et al. obtained jute fibres coated with silver ions [5]. Rac-Rumijowska described the possibility of introducing silver nanoparticles into the NMMO cellulose spinning solution [6]. The literature also describes more complicated systems in which cotton fabric was modified by the mixture of tetraethoxysilane (TEOS) and triclosan (TC)-(2,4,4′-trichloro-2′-hydroxydiphenyl ether) or TEOS and quaternary ammonium salt [7].

An interesting and modern material used to prepare polymer composites is graphene oxide (GO), which has many different oxygen-containing functional groups [8]. GO can be easily dispersed in water and organic solvents, e.g., DMF, tetrahydrofuran, and ethylene glycol [9,10]. The diversity of oxygen functional groups arranged on the GO surface makes it easy to be dispersed in polymer with functional groups to form durable bonds [11]. Thanks to its specific structure, graphene oxide has a number of interesting properties. The most important of them are bactericidal and fungicidal properties, used in many composite materials, such as fibres or membranes [12,13,14]. In general, the development of antimicrobial activity for GO proceeds in three steps: (1) Deposition of nanosheets on the bacterial surface, (2) membrane disruption by sharp nanosheets, and (3) the ensuing superoxide anion-independent oxidation [15,16]. The discussed bactericidal properties result from the interactions between GO and bacterial cells. These involve mechanical damage of cell walls and destruction of lipid layers, resulting in oxidative stress and, consequently, death of a cell [17,18]. The mechanism of the destruction of bacterial and fungal cells by GO is closely determined by such GO properties as: Concentration, particle size, oxygen group content, agglomerate size, chemical purity, and pathogen incubation time [19,20,21]. The results of the studies by other researchers also indicate a connection between biocidal properties and the surface charge determined on the basis of the surface zeta potential [22]. Thus, the biocidal properties of GO/CEL materials may depend on the surface charge and the isoelectric point value [23].

The literature widely describes various methods of obtaining cellulose composites with the addition of GO. Most reports in the literature concern composites in the form of cellulose membranes [24,25,26], granules [27,28], hydrogels [29], and aerogels [30] with the addition of GO. Unfortunately, there are few reports describing the use of GO for the production of fibres with biocidal properties. One of the simpler methods is coating cotton fabric with the GO dispersion [31]. Yang et al. coated amine-functionalized cellulose yarn with the GO dispersion [32]. Tian’s team produced cellulose composite fibres using the NaOH aqua solution method [33]. Teodoro et al. described the method of creating the GO/CEL nanofibers, which were used as mercury detectors [34]. Other researchers obtained bacterial cellulose in the presence of GO, producing a composite material [35,36,37].

This paper presents the results of research on cellulose composite GO/CEL fibres, obtained according to the procedure described in our earlier publication [38]. The fibres were formed by wet spinning method using 5% CEL solutions in ionic liquid: 1-ethyl-3-methylimidazolium acetate (EMIMAc) with GO/DMF addition. The process of fibre formation, consisting of extrusion spinning liquid into different baths: Water and methanol. The paper demonstrates the influence of GO additive on the structural and mechanical properties of the obtained composite fibres. It proves the relationship between the surface zeta potential and the isoelectric point, and the biocidal properties of GO/CEL fibres against Gram-negative bacteria (*Escherichia coli*), Gram-positive bacteria (*Staphilococus aureus*), and *Candida albicans* fungi. The obtained composite fibres based on CEL doped with GO may find potential applications in the widely understood medical industry.

## 2. Materials and Methods

### 2.1. Materials

Cellulose long fibres C 6663, 1-ethyl-3-methylimidazolium acetate (EMIMAc) 97% (*w/w*) purity, graphite powder <20 μm, and Triton X-100 were purchased from Sigma-Aldrich. Methanol (99.8%), KMnO_4_, H_2_SO_4_, with 98% (*w/w*) purity, 30% H_2_O_2_, *N,N*-dimethyloformamide (DMF) purchased from Avantor Performance Materials Poland S.A. (Gliwice, Poland). All the chemicals were used without further purification.

Graphene oxide was obtained according to modified Hummers method [39]. In brief, 1 g of NaNO_3_, 46 mL of H_2_SO_4_, and 2 g of graphite powder were placed in a flask in an ice bath. After stirring for 30 min, 6 g of KMnO_4_ was added in small portions so that the temperature of the mixture did not exceed 20 °C. After adding all KMnO_4_ and waiting for 5 min, the mixture was heated to 35 °C and stirred at this temperature for 4 h. Then, 92 mL of distilled water was carefully added in portions. Finally, the unreacted KMnO_4_ was removed. For this purpose, 80 mL of distilled water at 60 °C and 50 mL of 30% H_2_O_2_ were added. The obtained GO was centrifuged and washed several times with distilled water until the wash water reached pH 7. Wet GO was dried in an oven at 60 °C, turning into a brown solid. The resulting GO powder was dispersed in DMF using an ultrasonic bath to prepare the 2.1% *w/w* GO/DMF dispersion.

### 2.2. Preparation of Fibres

To prepare spinning solutions, suitable amounts of 2.1% GO/DMF dispersion were introduced into the ionic liquid (EMIMAc), which was followed by intensive stirring for 10 min and sonication for 30 min in an Inter Sonic 1S-1 ultrasonicator at 35 kHz. Suitable amounts of cellulose were introduced into the dispersion thus obtained such that the CEL concentration in each spinning solution was 5%. Then, each solution was mixed thoroughly using a rotary homogenizer and left to deaerate for 24 h. The deaeration process was based on the free removal of air bubbles that moved to the surface of the spinning solutions under atmospheric pressure. The amounts of the ingredients that were used in the preparation of the individual spinning solutions are the same and described in detail in our previous publication [38]. The percentage concentrations of CEL and GO content in individual fibres are shown in Table 1.

**Figure 1:**
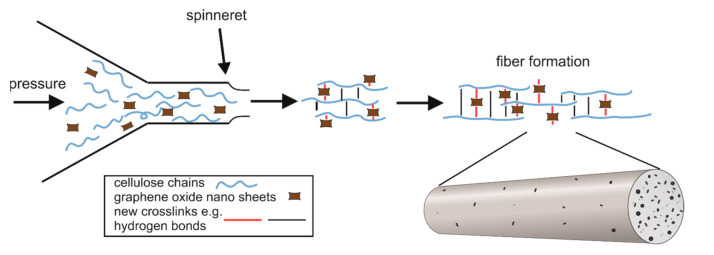
Diagram of the GO/CEL fibre forming process showing the distribution of GO particles in the cellulose matrix.

CEL and GO/CEL fibres were produced via the wet spinning solution method (Figure 1). For this purpose, a KDS-100 single syringe infusion pump (KD Scientific) that was equipped with a 10 mL syringe and a needle with an internal diameter of 0.7 mm was used. The fibre extrusion rate was 0.18 mL/min. The stretch ratio was S = 1. The monofilament take-up velocity was 23 cm/min. The fibres were extruded at room temperature directly into a bath that contained distilled water (1) or methanol (2). The coagulation process lasted 30 min, and subsequently, the fibres were taken up onto a spool and dried with air at 60 °C.

**Figure 2:**
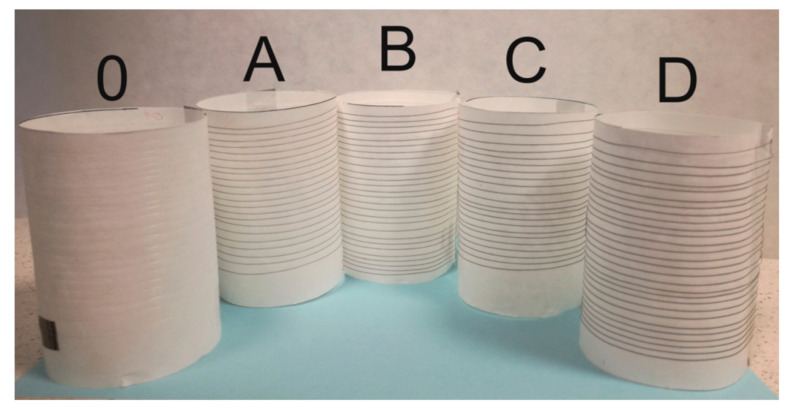
The photo shows pure cellulose fibres (**0**) and GO/CEL composite fibres (**A**–**D**). The obtained fibres look identical, regardless of the coagulation method (water-1; methanol-2).

We know from our own experience in working with GO/CEL membranes that cellulose composites may contain residual ionic liquid inside the material [25]. Therefore, the fibres obtained in the experiment were subjected to the washing process. The washing process of cellulose and nanocomposite fibres was carried out by using the home laundry washing method. The washing solution was prepared by dissolving 1 g of non-ionic surfactants Triton X-100 (Sigma-Aldrich, Poznan, Poland) in 400 mL of distilled water, and the fibres to solution weight ratio was 1:50. The fibres were immersed in a washing solution at 50 °C and mixed for 60 min. After washing, the fibres were rinsed two times in clean water and air dried (Figure 2).

### 2.3. Research Methods Used

The surfaces and cross-sections of the fibres were observed using an optical microscope (OPTA-TECH, Warsaw, Poland) at 10× magnification. Images of the sample were captured using transmission light mode.

Fibre tests were also conducted using a high-resolution Phenom ProX scanning electron microscope (SEM) from Thermo Fisher Scientific (Pik Instruments, Piaseczno, Poland) that was operated at 10 kV. Liquid nitrogen was used to prepare the cross-sections of the fibres, in which the samples were frozen and broken. The fibres’ thicknesses were measured from the surface images using FibreMetric software developed by PhenomWorld.

Nicolet 6700 FT-IR spectrometer (Thermo Electron Corp., Madison, WI, USA) equipped with a photoacoustic MTEC model 300 accessory was used in the FTIR (infrared spectroscopy) spectroscopic analysis. The following measurement parameters were used: Resolution, 4 cm^−1^; spectral range, 500–4000 cm^−1^; and number of scans, 64. Data collection and post-processing were conducted using the OMNIC software (v. 8.0, Thermo Electron Corp., Madison, WI, USA.).

Raman spectroscopy was performed with a Witec Raman Alpha M300+ spectrometer (WITec Corp., Ulm, Germany), with laser Nd-YAG at 532 nm, a laser power of approximately 1 mW, a spectral resolution of 2 cm^−1^, and a long working distance objective Olympus LMPLFLN 20X (Olympus Corp., Warsaw, Poland).

The zeta potential was measured using the streaming potential method applied to electrokinetic analyser SurPASS 3 (Anton Paar GmBH, Graz, Austria) according to Helmholtz-Smoluchowski equation Equation (1).

(1) $$\zeta =\frac{dU}{d\Delta p}\times \;\frac{\eta }{\varepsilon \;x\;\varepsilon_{0}}\times \;k$$

where: ***ζ*** is calculated zeta potential; ***U*** is measured streaming potential; ***Δp*** is pressure difference across the sample; ***η*** is viscosity of electrolyte solution; ***ε*_0_** is vacuum permittivity; ***ε*** is dielectric constant of the electrolyte; and ***k*** is electrolyte conductivity.

The zeta potential measurements were carried out in a cylindrical cell, into which fibre samples were introduced, then the cell was placed in the testing device and rinsed with electrolyte: 0.001 M KCl. Measurements of the zeta potential were carried out for the pH range of 3 to 10. Appropriate amounts of 0.1 M HCl and then 0.1 M KOH were dosed into the tested solution. Three washing cycles and four zeta potential measurements were performed for each pH value. During the tests, the isoelectric point (IEP) was also determined for each of the tested samples.

The strength parameters were determined by considering the recommendations of ISO 5079 (“Textiles fibres-Determination of breaking force and elongation of individual fibres (ISO 5079: 1995),” n.d.). The measurements were conducted using an Instron testing machine (Model 5544, Norwood, MA, USA) with a compression and stretching head with a measuring range of 0–10 N (“Technical-motion documentation of a resistance machine INSTRON,” n.d.). The tests were conducted at a strain rate of 10 mm/min for all samples. To determine the strength parameters, 50 ruptures were conducted for each variant, and a random error that was equal to 2% of the value of the average breaking force was assumed. The measuring distance between the jaws was 20 mm. The testing was conducted under normal climate conditions (“Textiles-Standard atmospheres for conditioning and testing (ISO 139:2005),” n.d.).

The microbiological studies were performed as follows. The specimens were exposed to bacteria and fungi capable of causing human infections, i.e., gram-positive Staphylococcus aureus, gram-negative Escherichia coli, and Candida albicans fungi. Microorganisms (reference strains) were purchased from the American Type Culture Collection (ATCC, Kielpin, Poland). The bacteria were grown on blood agar at 36 ± 2 °C for 24 h. Blood agar is an enriched culture medium. The medium is non-selective, used to grow anaerobic, aerobic, gram-negative, and gram-positive bacteria as well as fungi. The ingredient is agar and most frequently sheep’s blood. Candida albicans was grown on Sabouraud agar. Sabouraud medium is used in microbiology to grow fungi. Its basic ingredients include agar, distilled water, growth sources (glucose, peptone), and antibiotics. The latter most often include penicillin, streptomycin, or chloramphenicol, used to inhibit the growth of bacteria, as is the acidic pH. The grown cultures were rinsed with 1 mL of physiological NaCl saline solution. Using a sterile pipette, 0.1 mL of the microorganism suspension was drawn and transferred to selective agars by inoculating “spread plates”. In this case, the following media were used to cultivate the microorganisms: Chapman agar-*S. aureus*, MacConkey agar-*E. coli*, and Candida-*C. albicans*.

Chapman agar is a selective-multiplication medium used for the cultivation of staphylococci. It takes advantage of the fact that these bacteria are able to grow in high concentrations of sodium chloride. Chapman agar contains 7.5% NaCl solution (to inhibit the growth of bacteria other than staphylococci), growth sources (broth, peptone), mannitol, and phenol red. MacConkey Agar is a selective medium used in microbiology for the cultivation of gram-negative bacteria. The components that differentiate bacteria are: Lactose and pigments. The crystal violet contents inhibits the growth of gram-positive bacteria, while neutral red colours the lactose-fermenting microorganisms. MacConkey Agar differentiates gram-negative bacteria into lactose-fermenting (Lac+) and non-fermenting (Lac-). Those microorganisms that can ferment lactose contained in agar (e.g., *Escherichia coli*, *Klebsiella* sp.) acidify the medium to a pH value of <6.8. This leads to the formation of red-pink colonies. Lactose-negative bacteria such as Salmonella or Shigella consume peptones; they grow in the form of white/transparent colonies, because neutral red is colourless in neutral pH.

Candida agar, on the other hand, is used for the multiplication and quick identification of Candida yeasts. The cells of individual species, thanks to characteristic enzymes, break down the colour compounds present in the chromogenic mixture and by absorbing the dyes acquire different colours, which facilitates their identification. The selective factor of the medium, limiting the growth of bacteria, is chloramphenicol and lowered pH.

In the next stage of microbiological studies, samples of whole (1.5 cm long) and comminuted (0.5 mm long) GO/CEL fibres were placed on the central part of Petri dishes (with appropriate agars) (these fibres were test samples). The tests prepared in this way were placed in a laboratory incubator and incubated at the temperature of 36 ± 2 °C for 24 h. The experiment was performed three times for each type of composite fibre. A control sample (cellulose fibre 01 and 02) was also made. After the incubation process, the zones of inhibition of microorganism growth around the fibres were measured. The growth inhibition zones were analysed using optical microscope equipped with a camera (Opta-Tech, Warsaw, Poland).

## 3. Results and Discussion

### 3.1. General Characteristics of Fibres

The paper describes a method of obtaining fibres from pure cellulose and cellulose-based composite fibres with the addition of GO. Fibres were obtained using wet method from cellulose solution in EMIMAc, by coagulation in water (1) or methanol (2).

The molecular structure of the fibres surface was investigated using the FTIR spectroscopy. FTIR studies performed for the fibre samples after the washing process allowed for the evaluation of the ionic liquid removal. The spectra of pure cellulose and composite fibres did not differ from the spectra obtained for the CEL and GO/CEL membranes described in our earlier publication [40]. The results of the study are summarized in Table 2.

FTIR spectroscopy (Table 2) indicated that the fibres obtained by coagulation in water (1) and in methanol (2) did not differ in their chemical structure and contained the same bands. The characteristic wide band (3700 ÷ 2400 cm^−1^) of the elastic O–H indicating the formation of hydrogen bonds between GO and CEL. The GO addition probably causes loosening of the structure between cellulose macromolecule chains and introduces additional hydroxyl groups. For GO/CEL composite fibres, band intensities are observed at a wavelength of approximately 1640 cm^−1^ corresponding to the vibration of C=O groups in GO along with the amount of GO introduced to the cellulose fibres [41]. However, the 1404 cm^−^^1^ band, which is characteristic for this ionic liquid, was not observed [42]. The absence of this band was confirmed through the removal of EMIMAc from the fibres in the washing process. Our studies showed no significant shifts or changes in the peaks, indicating the incorporation of GO probably did not result in any change in the chemical structures of cellulose [43]. To investigate the interaction between cellulose and GO, Raman spectra of the GO and GO/CEL were performed. The GO displayed two characteristic peaks at ~1315 cm^−1^ (D peak) and ~1590 cm^−1^ (G peak), respectively. No shift was observed in the GO/CEL composite like in other publications [44].

**Figure 3:**
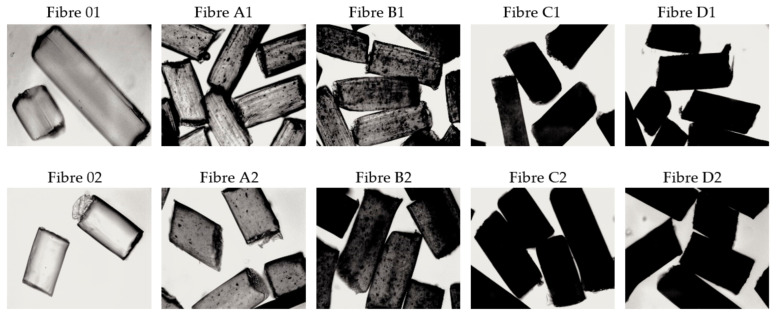
Optical microscope images of the surfaces of fibres that were coagulated in water (1) and in methanol (2) (10× magnification).

The method of GO dispersion in the CEL matrix influences physicochemical, structural, and functional properties of composite fibres. Optical microscopy allows a quick analysis of the structure of the examined fibres (Figure 3). The pure cellulose fibres (01; 02) are smooth and transparent in the longitudinal view. Transmission mode optical microscopy enabled the observation of the presence of GO inclusions in GO/CEL composite fibres (Figure 3). The more GO (0.21; 0.5; 0.98; and 1.97 % *w/w*) was added into the GO/CEL fibre, the darker the sample. GO is well dispersed throughout the entire volume of the GO/CEL composite.

**Figure 4:**
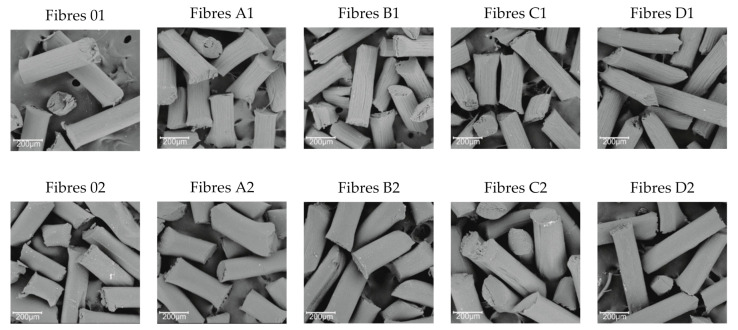
SEM (scanning electron microscopy) images for surfaces fibres that were coagulated in water (1) and methanol (2) (300× magnification).

SEM microscopy (Figure 4) allowed us to analyse the surface of pure cellulose fibres (0–1; 0–2) and GO/CEL fibres coagulated in water (1) and in methanol (2). The pictures show that all the fibres coagulated in methanol (2) in general have a smooth surface and fine efflorescence. The more GO in composite fibres (2), the less surface efflorescence and more shallow cracks along the fibre. On the other hand, the fibres coagulated in water (1) show numerous grooves extending along the fibre. The more GO in the fibres (1), the deeper and more numerous the cracks on the surface. The surface morphology of the examined fibres is the result of the fibre coagulation method. The use of methanol results in almost immediate precipitation of the fibres in the coagulation bath. The coagulation of the fibres in water, in turn, is a slower process, and the slow penetration of the fibre can lead to the formation of a porous structure. We described similar observations for GO/CEL composite membranes, examining the effect of the coagulant and the amount of GO additive on their porosity [40].

**Figure 5:**
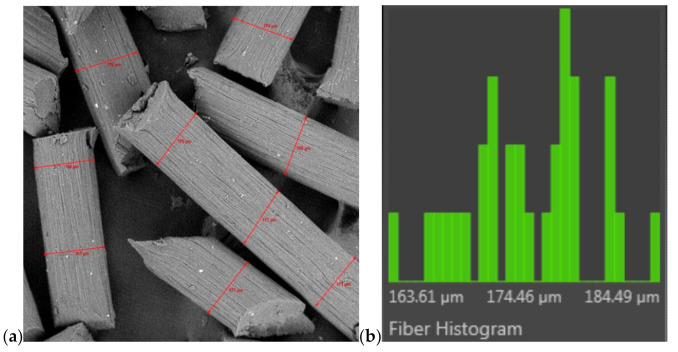
A measurement of thickness fibres by using the FibreMetric software; (**a**) surface of fibres (300× magnification) and (**b**) histogram (an example for fibres C1).

The diameter of all obtained fibres before (nw) and after the washing process was measured using the FibreMetric software. An example of tests on a selected sample is shown in Figure 5, and the obtained results of thickness measurements are summarized in Table 3.

The process of coagulation of fibres in methanol (Table 3-not washed fibres “nw”) is relatively fast. The polarity of this solvent is 1.7 [D], which accelerates the coagulation process of 02 fibres. On the other hand, the coagulation of 01 fibres in water is closely related to the polarity of the water molecule, which is 1.85 [D], slowing down the process of washing away the ionic liquid. The effect of these interactions is the thickness of CEL fibres, which is: 156 µm and 183 µm (for 02nw fibres and 01nw fibres), respectively. The thicknesses of GO/CEL fibres (Table 3-not washed fibres “nw”), on the other hand, have slightly different values, which may be related to interactions at the molecular level. Thus, a general conclusion can be drawn that the more GO additive, the more interactions between CEL and GO and between GO nanoparticles themselves. The discussed interactions were observed as a broad band of 3400–2400 cm^−1^ (Table 2). While interactions between GO nanoparticles, which resulted in the formation of agglomerates, were observed in Figure 3.

When analysing the results of the fibres’ thickness measurements after the washing process (Table 3-fibres after washing), it can be observed, as a general tendency, that this process reduces the thickness of all the tested fibres. During washing, individual fibres lose 7–15% of their thickness. The observed phenomenon is probably the result of the removal of the ionic liquid (EMIMAc) from the inside of the fibres, which was confirmed by FTIR studies (Table 2).

Some regularities can be observed when comparing the thicknesses of all the composite fibres listed in Table 3. For samples A1, B1, A2, and B2, a slight increase in thickness is observed as the amount of GO addition increases. On the other hand, the thickness of the 1.97% GO fibres is lower than in the C1 and C2 samples. This phenomenon is probably the result of an excess of GO addition, which causes the formation of large amounts of agglomerates in samples D1 and D2.

The obtained results prompted us to analyse the influence of the fibre formation process, the composition of the spinning solution, and the washing process on the mechanical properties of the produced CEL and GO/CEL fibres.

First, elongation at break was analysed for all fibres tested before the washing process (Table 4—fibres before washing). The studies have shown that for the fibres coagulated in water (1), the elongation at break values are low. Apart from this, there are no significant dependencies between elongation at break for individual fibre samples. Elongation at break for pure cellulose (01nw) was ~10%, for composite fibres (A1nw-C1nw) it was ~7–~14%, while for sample D1nw it had the lowest value of ~5%. For composite fibres obtained by the second method (2), there is a clear increase in elongation at break to the value of ~20% for A2nw-C2nw fibres, i.e., fibres containing 0.21–0.98% *w/w* of GO. On the other hand, for the D2nw fibres and for the 02nw fibres, the elongation at break values are similar, i.e., ~10%. Therefore, it can be concluded that the addition of 1.97% w/w of GO to the cellulose matrix is too much.

The same studies were carried out for the samples of washed fibres (Table 4—fibres after washing). It turned out that the washing process increases elongation at break by about 80% for the fibres coagulated in methanol (02). For fibres 01, the increase was also present, but at ~30%. The obtained results are higher than those described in the work by Cao et al. [45]. However, for all composite fibres, a decrease in elongation at break as a function of increasing amount of GO was noted. All values of elongation at break for CEL and GO/CEL fibres fall within the range characteristic for commercial cellulose fibres, i.e., 6–10% [46].

Analysing the tenacity results for GO/CEL fibres (Table 4), it is evident that the fibre samples coagulated in methanol (2)—both not washed and washed—generally have higher tenacity values as compared to the corresponding samples of fibres coagulated in water (1). Moreover, for all series of composite fibres, the tenacity increase is observed as the GO content rises to 0.5% *w/w*, then it decreases with its lowest values for samples containing 1.97% of GO. The observed tenacity changes are similar to the changes in the thickness of the examined fibres (Table 3). These studies show that during the process, a reconstruction of the internal structure takes place, accompanied by an increase in fibres tenacity and elongation at break. Comparing the obtained results with the literature data [46], it can be concluded that the obtained fibres have good tenacity, higher than that of regenerated cellulose, which is 69–170 [MPa].

The next area of study was determining the elastic modulus (Table 4). This parameter has higher values for all samples of unwashed fibres (nw), and after the washing process, all values of the elastic modulus drop sharply, e.g., by 83% for fibres 02 and 93% for fibres C2. Therefore, it can be assumed that during washing, the components responsible for fibre stiffness are washed out of all the tested fibres. The elastic modulus values for GO/CEL fibres do not depend on the coagulant (water or methanol) used in the fibre formation process. However, they are slightly dependent on the amount of GO addition in the cellulose matrix. As the content of GO in unwashed fibres (nw) increases, so do the values of the elastic modulus. On the other hand, the opposite is true for unwashed fibres. The most interesting case are fibres containing 0.5% of GO additive in the cellulose matrix. In all tests of their mechanical properties, the results of B1 and B2 differ from the others, which may indicate that these composite fibres are characterized by the best quantitative composition.

### 3.2. Biocidal Properties of GO/CEL Fibres

**Figure 6:**
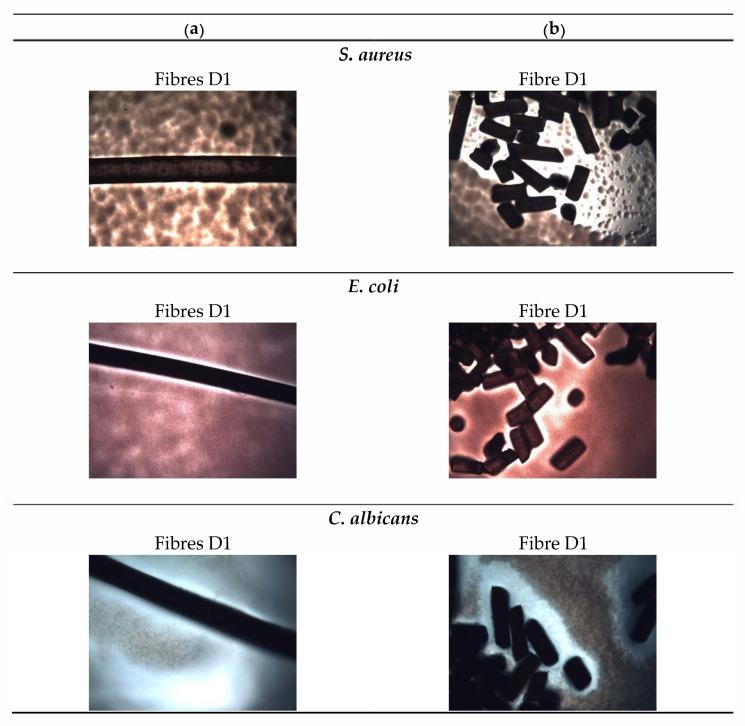
Photographs of microbiological tests of fibres with the highest amount of GO (D1). The photos show the tests for samples of: (**a**) Whole fibres; (**b**) fibre sections. The photos were taken after 24 h of *S. aureus*, *E. coli*, and *C. albicans* incubation.

Microbiological studies were carried out for both GO/CEL fibres and for the corresponding pure cellulose fibres. The following human pathogens were selected for the study: Gram-negative bacteria (E. coli), gram-positive bacteria (S. aureus), and fungi (C. albicans), and the sample photos taken with an optical microscope are presented in Figure 6.

**Figure 7:**
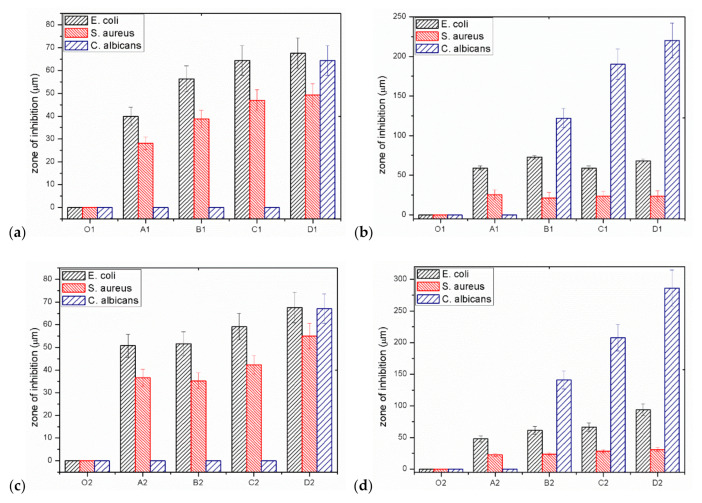
Results of the inhibition zone measurements for individual micro-organisms: (**a**) Whole fibres; (**b**) short sections of water coagulated fibres (1); (**c**) whole fibres; (**d**) short sections of fibres coagulated in methanol (2).

At the beginning, the impact of composite fibres on the multiplication or stopping the growth of Gram-negative *E. coli bacteria* was analysed. For GO/CEL fibres, the size of the *E. coli* inhibition zones depends on the form of the test sample. In general, samples coagulated in water A1–D1 and tested in the form of whole fibres (Figure 7a) have inhibition zones ranging from ~39.9 µm to ~64.4 µm, which increase as the concentration of GO in the fibres increase. The same fibres, but prepared in the form of small sections (Figure 7b), are characterized by slightly larger inhibition zones, which range from ~59.2 µm to ~72.8 µm. The test results obtained for GO/CEL fibres coagulated in methanol (2) A2–D2 were similar. The fibres tested as a whole (Figure 7c) were characterized by inhibition zones in the range of ~50.7–~67.6 µm. Whereas the same fibres, but cut into small lengths (Figure 7d), demonstrated the inhibition zones from ~47.9 µm to ~93.9 µm. It could, therefore, be concluded that the inhibition of *E. coli* multiplication is more intense if the GO/CEL fibre samples are cut into pieces.

Another tested microorganism was the gram-positive bacteria, *S. aureus*. Additionally, in this case, the size of the inhibition zones of *S. aureus* depends on the form of the test sample. Samples coagulated in water A1–D1, tested in the form of whole fibres (Figure 7a), are characterized by inhibition zones from ~28.2 µm to ~49.3 µm. The same samples, but in the form of staple fibres (Figure 7b), have smaller inhibition zones ranging from ~21.1 µm to ~25.4 µm. On the other hand, the test results obtained for the GO/CEL fibres coagulated in methanol (2) A2–D2 showed that the fibres tested as a whole (Figure 7c) were characterized by inhibition zones range of ~35.2–~47.0 µm. Whereas the same fibres, but cut into small lengths (Figure 7d), demonstrated slightly smaller inhibition zones ranging from ~23.5 µm to ~31.0 µm. The conclusion of the studies would be that the inhibition of *S. aureus* multiplication is more intense on the surface of GO/CEL fibres.

The last microorganisms used were fungi, *C. albicans*. The results of the tests presented in Figure 7a,c show that the fungus multiplication stops when the composite fibre samples (D1, D2) contain the highest amount of GO addition (1.97% *w/w*). For samples of fibres cut into sections (Figure 7b,d), on the other hand, the degree of stopping the pathogen multiplication increases with the increase in the content of the GO additive. In the studies of fibres coagulated in water (Figure 7b), the inhibition zone of *C. albicans* is high, ranging from ~122.1 µm to ~220 µm, and includes samples containing 0.5–1.97% *w/w* of GO. While for fibres coagulated in methanol (Figure 7d), the degree of inhibition is in the range of ~140.9 µm to ~286.4 µm for fibre samples: B2; C2; D2. It should therefore be concluded that the inhibition of *C. albicans* multiplication is more intense if GO/CEL fibre samples are cut into pieces and contain at least 0.5% of GO in the cellulose matrix.

Measurements of the microorganism inhibition zones (Figure 7) showed that even a small addition of GO results in a biocidal effect. The greater the effect is, the higher the concentration of GO in the tested fibres. In contrast, for the reference fibre samples (01 and 02), as expected, no inhibition zones were found. An interesting phenomenon is also the divergence in the width of the inhibition zones between the fibre samples with the same compositions, but a different coagulant. These differences may result from the structure of the fibre surface as observed using SEM in Figure 4. All fibres coagulated in methanol have a smooth surface, and those coagulated in water have fine grooves, which may hinder the access of bacteria to their surface.

The general conclusion of the microbiological studies is that for all GO/CEL fibres, the size of the inhibition zones depends on the method of sample preparation and takes the highest values for fibres cut into sections (Figure 7b,d). The explanation for this phenomenon can be found in the anatomical structure and properties of individual microorganisms.

**Figure 8:**
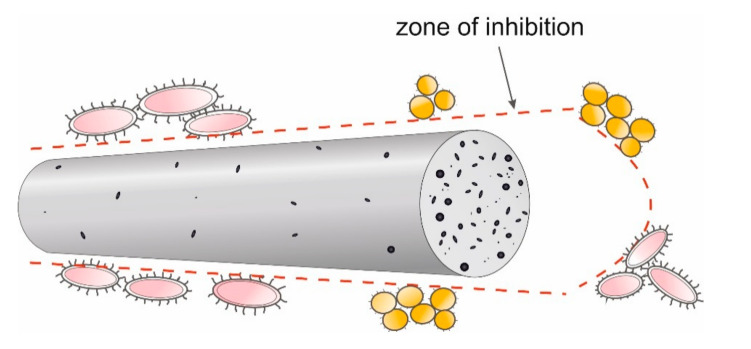
Model of pathogen cell destruction on the surface and at the cross-section of the fibre.

*E. coli* have a rod shape of 2 × 0.8 µm and are composed of a thin cell membrane. The shape of the bacterial allows it to adhere to the surface and to the cross-section of the GO/CEL fibre (Figure 8). The microscopic studies (Figure 3) show that GO particles are present on the surface of composite fibres in a small amount only. However, smaller and larger clusters of these nanoparticles occur inside of the fibres. The knowledge of the structure of GO/CEL fibres and the structure of *E. coli* cells allows us to understand the phenomenon of stopping the growth of this pathogen. The smaller inhibition zone observed in Figure 7a,c results from the presence of GO that is lower on the surface than inside the fibre (Figure 7b,c).

*S. aureus* bacteria, on the other hand, are spherical in shape, with dimensions of 1 × 0.8 µm and a thick cell wall. All these factors make the pathogen difficult to be destroyed by GO nanoparticles, both on the surface and in the cross-sections of GO/CEL fibres (Figure 8). This results in smaller inhibition zones that are observed in Figure 7.

*C. albicans* is a fungus which in the presence of sugars—in our case cellulose—forms a biofilm that sticks tightly to the surface of the fibre. Only a large amount of GO in the cellulose matrix inhibits its development. Therefore, the surfaces of composite fibres containing 1.97% of GO (Figure 7a,c), and the cross-sections of fibres containing 0.5% to 1.97% of GO, are resistant to *C. albicans* (Figure 7b,d).

The conducted microbiological tests clearly indicate the bactericidal effect of GO. Its mechanism can be explained in two ways. The first one concerns the mechanical damage to the bacterial cell walls, which in turn leads to metabolic disorders, oxidative stress, and cell death. This process is widely described by many researchers, among others in [47,48]. The second mechanism of GO influence on microorganisms is relatively little known. It consists in introducing surface functional groups that change the surface charge properties of materials. The functional groups in GO are hydroxyl and carboxyl groups, which determine its negative charge (zeta potential ~39 mV). This affects the surface charge parameter and, consequently, the isoelectric point (IEP) [22]. The experimental works described in the literature indicate the link of IEP with antimicrobial activity [23], therefore we analysed the zeta potential of the GO/CEL fibre surface.

**Figure 9:**
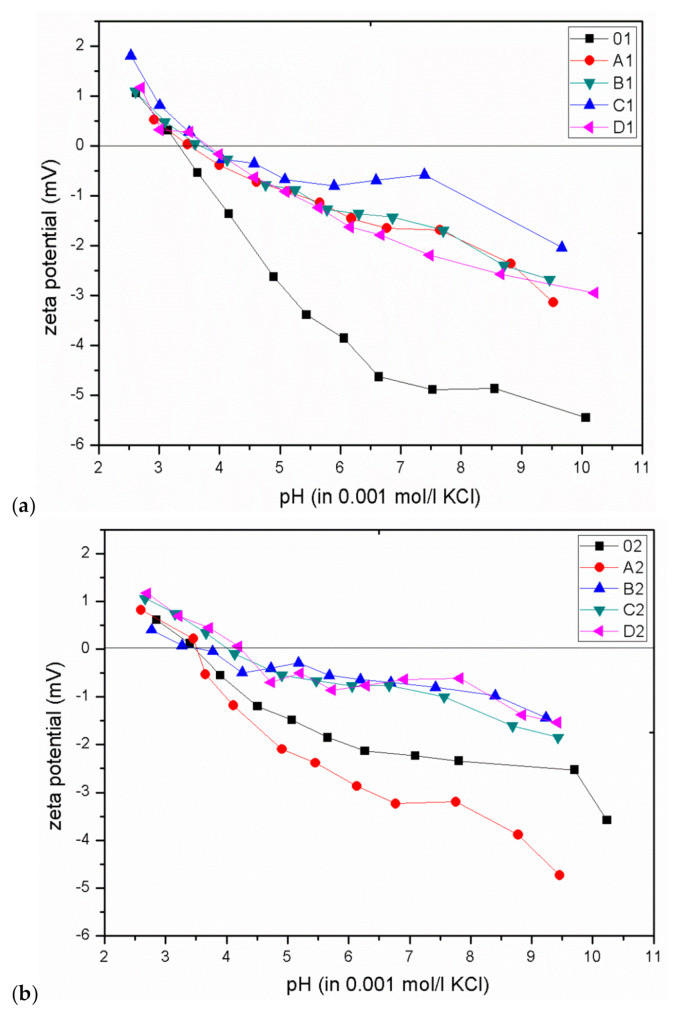
The dependence of the zeta potential on the pH value for CEL and GO/CEL fibres coagulated in water (**a**) and methanol (**b**). Straight line designates the isoelectric point (IEP) (where ζ = 0 mV).

The dependence of the degree of bactericidal properties on the zeta potential was determined based on the isoelectric point (IEP) of the CEL and GO/CEL fibres. For this purpose, measurements of the zeta potential of all obtained fibres were performed in the pH range of 3 to 10, and the results are summarized in Figure 9. Pure CEL fibres, as compared to composite fibres, show a more negative zeta potential value, which results from the large number of hydroxyl groups present in cellulose. The addition of GO (which also has numerous oxygen groups) to the cellulose matrix causes the formation of hydrogen bonds between cellulose and graphene oxide. As a result, as the concentration of GO in the fibres increases, the acidic nature of the GO/CEL fibres decreases, which is expressed by a change in the shape of the ***ζ*** = f (pH) curve into a more inert one. It is related to the shift of the IEP towards higher pH values and the “flattening” of the curve [49].

**Figure 10:**
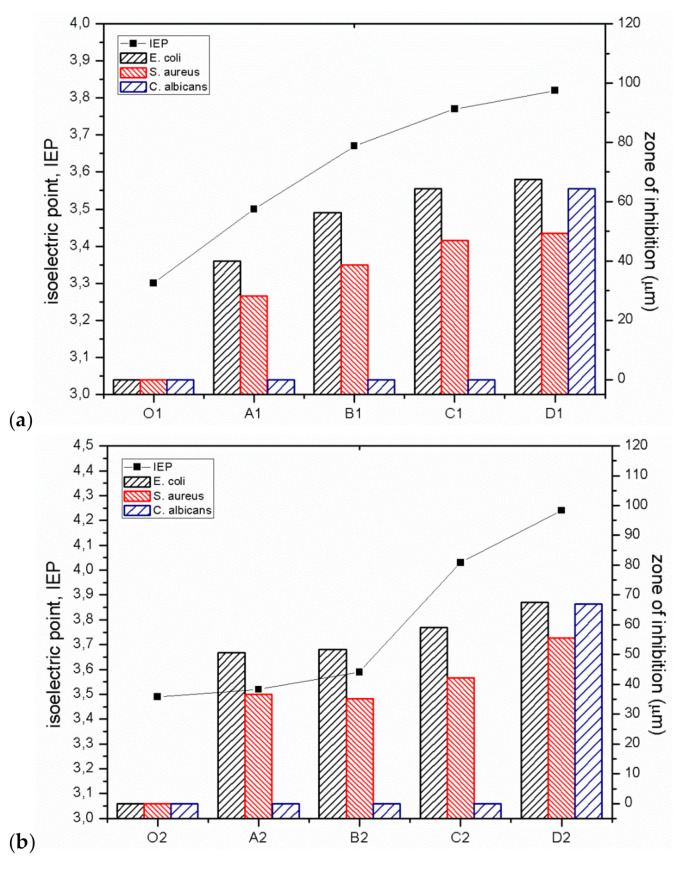
Antimicrobial activity (shown as the zone of inhibition) and IEP of CEL and GO/CEL fibres coagulated in water (**a**) and coagulated in methanol (**b**) on bacteria *E. coli*, *S. aureus*, and fungi *C. albicans*.

The bactericidal properties of GO/CEL fibres mainly result from the increase in the IEP value, which is confirmed by the work of other researchers [22,23]. Analysis of the our research shows that for fibres coagulated in water (01; A1; B1; C1; D1), the IEP values are within in the range of 3.3–3.8. On the other hand, for fibres coagulated in methanol (02; A2; B2; C2; D2), the IEP values range from 3.5 to 4.2. In both cases, the IEP values increase with the increase of GO concentration in composite fibres. The relationship between the IEP value and the size of the pathogen inhibition zones for all obtained fibres is shown in Figure 10.

The results of the research indicate a close correlation between the IEP value and bactericidal properties (Figure 10), both in the case of gram-negative (*E. coli*) and gram-positive (*S. aureus*) bacteria. It was observed that an increase in IEP results in increased bactericidal activity. This relationship is very well visible for fibres coagulated in water (Figure 10a), but also in the case of fibres coagulated in methanol (Figure 10b). The reduction of the negative surface charge of composite fibres (Figure 9) and the higher value of the zeta potential are accompanied by the bactericidal effect. This effect is caused by the formation of hydrogen bonds and electrostatic interactions between the surface of GO/CEL fibres and the bacterial cell wall, which prevents the absorption of nutrients and causes cell death [15,50]. Therefore, electrostatic interactions between GO/CEL fibres and the negative surface charge of bacteria are possible, which inhibits the growth of bacteria [51].

## 4. Conclusions

A simple method of obtaining composite fibres containing a wide range of concentrations of GO (from 0.21% to 1.97%) in the CEL matrix was developed. The method enables good dispersion of the nano-additive in the polymer matrix, which was confirmed by optical microscopy. The addition of nanoparticles also influenced the mechanical properties of the formed GO/CEL fibres. Composite fibres were characterized by high elongation at break and tenacity. FTIR studies enabled us to observe the formation of GO-CEL intermolecular hydrogen bonds. An additional feature of GO/CEL fibres are biostatic properties, confirmed by the study, preventing the development of bacteria (*E. coli* and *S. aureus*) and fungi (*C. albicans*) on their surface and cross-section. The size of the inhibition zones is proportional to the concentration of GO in the fibres. The zeta potential study allowed us to determine the surface charge and IEP values, the increase of which is responsible for the increased biocidal effect of GO/CEL fibres. The obtained composite fibres based on CEL doped with GO may find potential applications in the widely understood medical industry.

## Figures and Tables

**Table 1:** Percentage concentrations of cellulose fibres (CEL) and graphene oxide (GO) in individual fibres.

Determination of the Obtained Fibres	0	A	B	C	D
*W/w* conc. of GO in fibre [%]	0	0.21	0.50	0.98	1.97
*W/w* conc. of CEL in fibre [%]	100	99.79	99.50	99.02	98.03

**Table 2:** Summary of the most important bonds appearing in the infrared spectroscopy (FTIR) spectra of CEL and GO/CEL fibres.

**Samples**	The FTIR Peaks (cm^−1^) *	**Bond Type**
All fibres	3400–2400	Wide band of the elastic O–H vibrations in the hydrogen bonds
All fibres	2900	Stretching vibrations of the C–H oscillator
Composite fibres	1645 (1); 1637 (2)	Vibration of C=O groups in GO
All fibres	1160	Asymmetric stretching vibrations of C–O–C in the pyranose ring
All fibres	1115 (1); 1117 (2)	Oscillation of etheric C–O–C groups between the pyranose rings

Where *: (1)—fibres coagulated in water; (2)—fibres coagulated in methanol.

**Table 3:** Summary of the median values of results of the fibre thickness measurements.

**Fibre Thickness (before Washing) (µm)**									
**01nw**	**A1nw**	**B1nw**	**C1nw**	**D1nw**	**02nw**	**A2nw**	**B2nw**	**C2nw**	**D2nw**
183 ± 6	175 ± 6	174 ± 8	174 ± 5	165 ± 5	156 ± 14	182 ± 7	187 ± 8	186 ± 8	180 ± 7
**Fibre Thickness (after Washing) (µm)**									
**01**	**A1**	**B1**	**C1**	**D1**	**02**	**A2**	**B2**	**C2**	**D2**
169 ± 6	151 ± 5	156 ± 7	148 ± 5	147 ± 7	141 ± 7	163 ± 12	165 ± 4	165 ± 8	150 ± 9

nw—sample not washed.

**Table 4:** Mechanical test results.

Fibres before Washing [38]				Fibres after Washing			
	E (%) *	W (MPa) *	M (GPa) *		**E (%)**	**W (MPa)**	**M (GPa)**
01nw *	10.01 ± 2.37	137.22 ± 34.36	35.67 ± 3.06	**01**	13.05 ± 0.92	223.61 ± 13.28	7.91 ± 0.07
A1nw *	12.79 ± 0.53	149.35 ± 6.66	59.00 ± 3.88	**A1**	11.9 ± 1.37	188.92 ± 25.87	7.41 ± 0.45
B1nw *	7.44 ± 1.01	165.86 ± 13.91	62.69 ± 3.55	**B1**	9.5 ± 1.45	219.70 ± 20.06	8.52 ± 0.59
C1nw *	14.32 ± 1.10	95.80 ± 4.38	48.92 ± 6.57	**C1**	8.75 ± 0.51	132.75 ± 11.11	5.30 ± 0.51
D1nw *	5.12 ± 0.49	111.38 ± 6.78	70.30 ± 4.55	**D1**	7.45 ± 0.77	167.26 ± 9.23	6.97 ± 0.70
02nw *	10.87 ± 2.21	88.06 ± 25.18	35.99 ± 4.02	**02**	10.25 ± 1.90	228.67 ± 28.05	8.81 ± 0.91
A2nw *	20.06 ± 4.51	202.11 ± 25.27	53.70 ± 4.09	**A2**	8.55 ± 1.34	287.32 ± 20.01	10.71 ± 0.82
B2nw *	19.54 ± 3.93	224.33 ± 15.72	83.34 ± 3.89	**B2**	7.7 ± 1.41	172.16 ± 13.75	6.56 ± 0.55
C2nw *	19.93 ± 1.41	154.54 ± 6.20	92.51 ± 6.30	**C2**	7.65 ± 1.01	189.65 ± 15.21	7.41 ± 0.69
D2nw *	10.27 ± 1.61	146.31 ± 8.46	81.91 ± 3.49	**D2**	19.55 ± 1.25	193.41 ± 21.09	6.25 ± 0.69

where: *—is nw-not washed sample, E—elongation at break; W—tenacity; M—elastic modulus.
